# Correction: *In situ* growth of TiO_2_/SiO_2_ nanospheres on glass substrates *via* solution impregnation for antifogging

**DOI:** 10.1039/c9ra90089f

**Published:** 2019-12-04

**Authors:** Fang Liu, Jie Shen, Wuyi Zhou, Shiying Zhang, Long Wan

**Affiliations:** College of Materials Science and Engineering, Hunan University Changsha 410082 PR China wanlong1799@163.com +86-731-84261297; Hunan Key Laboratory of Applied Environmental Photocatalysis, Changsha University Changsha 410022 PR China; College of Materials and Energy, South China Agricultural University Guangzhou 510642 China

## Abstract

Correction for ‘*In situ* growth of TiO_2_/SiO_2_ nanospheres on glass substrates *via* solution impregnation for antifogging’ by Fang Liu *et al.*, *RSC Adv.*, 2017, **7**, 15992–15996.

The Acknowledgements section should be corrected as follows (an incorrect funding was cited):

We are grateful for the financial support from the National Natural Science Foundation of China (51272032), the Hunan Provincial Natural Science Foundation of China (14JJ5010), the Hunan Provincial Innovation Foundation for Postgraduate (CX2016B068) and the Aid Program for Science and Technology Innovative Research Team in Higher Educational Institutions of Hunan Province.

The Royal Society of Chemistry apologises for these errors and any consequent inconvenience to authors and readers.

## Supplementary Material

